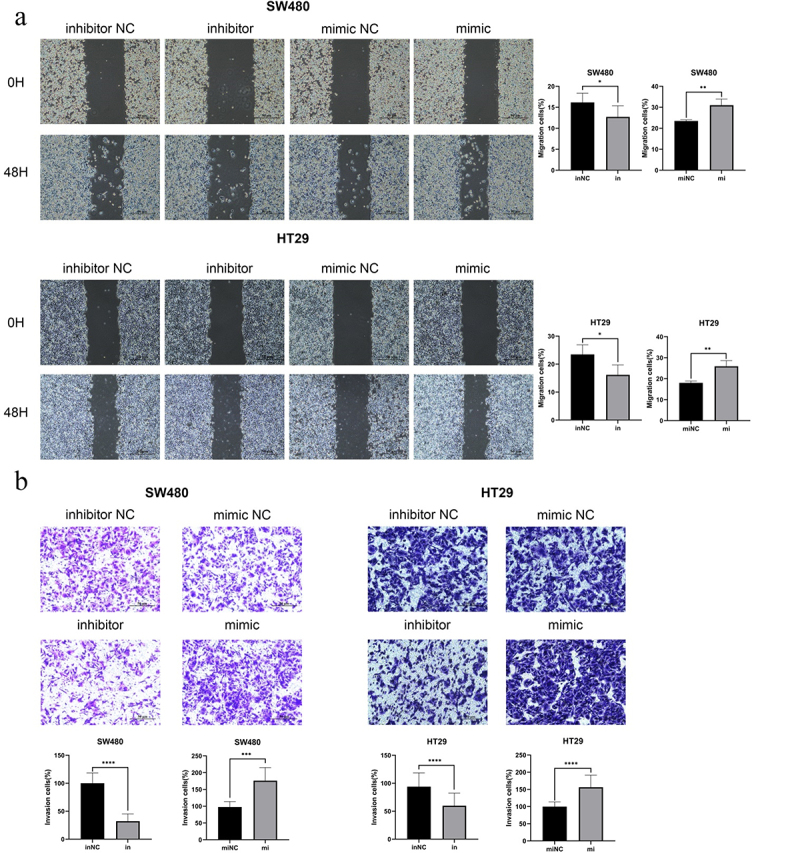# Correction

**DOI:** 10.1080/15384047.2024.2389605

**Published:** 2024-08-11

**Article title: MiR-135b-5p promotes cetuximab resistance in colorectal cancer by regulating FOXN3**

**Authors**: Chun Peng, Xiaoqing Li,Yuhui Yao, Yu Nie, Lingyao Fan and Chuandong Zhu

**Journal**: Cancer Biology & Therapy

**DOI**: https://doi.org/10.1080/15384047.2024.2373497

The authors recently noticed that in this article, the images of Figure 1 and 3 were inadvertently misplaced during the preparation of these figures. Figure 1 and 3 are not supposed to be that one presented in the article. The amended version of the figures are now shown below.

**Figure 1:**
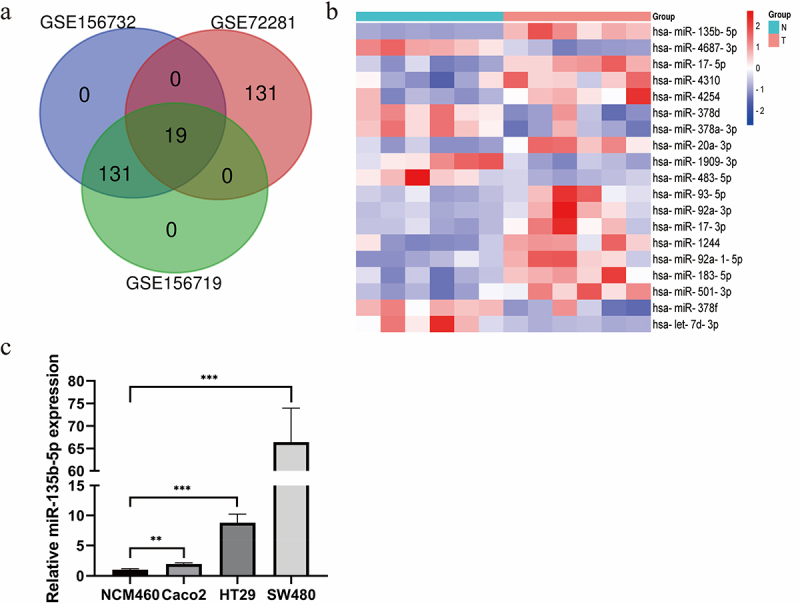

**Figure 3:**